# Late diagnosis of respiratory syncytial virus and influenza co-infection during coronavirus disease 2019 pandemic: a case report

**DOI:** 10.1186/s13256-023-04187-3

**Published:** 2023-10-21

**Authors:** Elham Barahimi, Mehdi Hassani Azad, Zahra Ghaeini Hesarooeyeh, Niloofar Hosseini Hafshejani, Sahar Defaee, Narjes Seddighi

**Affiliations:** 1 Infectious and Tropical Diseases Research Center, Hormozgan Health Institute, Hormozgan University of Medical Sciences, Bandar Abbas, Iran; 2 Student Research Committee, Faculty of Medicine, Hormozgan University of Medical Sciences, Bandar Abbas, Iran; 3 Internal Medicine Department, Shahid Mohammadi Hospital, Hormozgan University of Medical Sciences, Bandar Abbas, Iran; 4 Student Research Committee, Faculty of Para-Medicine, Hormozgan University of Medical Sciences, Bandar Abbas, Iran

**Keywords:** RSV, Influenza, COVID-19, Case report

## Abstract

**Background:**

Respiratory syncytial virus (RSV)-induced disease is one of the important causes of flu-like illness in older adults and can cause serious disease in those who are at high-risk medical conditions. During coronavirus disease 2019 (COVID-19) pandemic, because of overlapping symptoms of severe acute respiratory syndrome coronavirus 2 (SARS-COV-2) infection with other respiratory infections, diagnosing diseases based on clinical and radiological findings was challenging and could cause misdiagnosis.

**Case presentation:**

An 87-year-old Persian man was admitted to the hospital due to loss of consciousness, respiratory distress, tachypnea, and oliguria. He had previously hospitalized because of cough, fever, loss of appetite, and fatigue. Severe acute respiratory syndrome coronavirus 2 (SARS-CoV-2) polymerase chain reaction (PCR) test was performed which was negative; however, based on ground glass opacity on his chest computed tomography (CT) scan and being on the outbreak of COVID-19, he fulfilled case definition of COVID-19; therefore, he received protocol’s treatment (remdesivir) for COVID-19 and relatively recovered and discharged. In our center, we requested brain and chest CT scans, blood tests, and multiplex PCR. Multiplex PCR revealed co-infection of influenza virus and RSV. Although we had started pneumonia and sepsis treatment, old age, weak immune system and the delay in initiation of right antibiotic and antivirus therapy altogether led him to die.

**Conclusion:**

As a takeaway lesson of this case report, it is necessary to pay attention to viruses that show similar symptoms during future specific virus pandemics, especially in patients with old age and weak immune systems.

## Background

Respiratory system infections are among leading causes of mobidity and mortality in children and the elderly. Influenza virus and respiratory syncytial virus (RSV) are two major pathogens of the respiratory system that can cause infection at any age [1].

RSV is the second leading cause of hospitalization from respiratory infections in elderly [2] which can cause upper respiratory system infection, pneumonia, and otitis media. Moreover, it is also the most common cause of bronchitis [1, 3, 4].

The disease caused by RSV is often mild; however it can cause serious disease in those who are at high-risk medical conditions such as cardiopulmonary disease [2, 3]. This disease is one of the important causes of flu-like illness in older adults [2].

The influenza virus infects respiratory epithelial cells [1]. Its symptoms usually appear suddenly, including fever, body aches, runny nose, muscle pain, weakness, and respiratory symptoms such as sore throat, cough, and rhinitis [5, 6].

Previous studies showed that simultaneous involvement with several respiratory infections is common; infection with two viruses at the same time can affect both the host (human) and each virus, moreover, have a great deal on the pathogenesis of the disease; the response of the immune system and treatment [1].

The results of an animal study on mice infected with RSV and/or influenza A virus (IAV) showed that simultaneous infection with these viruses increased the resistance of the respiratory tract and reduced thoracic compliance [1].

In this case, we introduced a patient with co-infection of influenza virus and RSV, who was wrongly diagnosed with coronavirus disease 2019 (COVID-19) at the beginning of his hospitalization.

## Case presentation

An 87-year-old Persian male patient, previously farmer, who was bedridden because of a hip fracture since 2 years ago, was admitted to Shahid Mohammadi hospital, a tertiary care center in the south of Iran, due to loss of consciousness (LOC), respiratory distress, tachypnea, and oliguria.

He had a history of 10 days of hospitalization in another hospital due to fever and cough. In that center, a severe acute respiratory syndrome coronavirus 2 (SARS-CoV-2) polymerase chain reaction (PCR) test was performed for the patient which was negative, however, based on ground glass opacity on his chest computed tomography (CT) scan and being on the outbreak of COVID-19, he fulfilled case definition of COVID-19; therefore, he received protocol’s treatment; remdesivir 200 mg stat and 100 mg daily for 5 days plus dexamethasone 6 mg daily intravenously. After relative recovery, he was discharged. The patient had no history of any chronic disease and did not receive any medications.

He had a history of weakness, fatigue, and loss of appetite which he received serum therapy in an outpatient clinic.

In our center, he was ill and toxic. His Glasgow Coma Scale (GCS) was seven.

Physical examination revealed heart rate = 107 beats per minute, blood pressure = 100/60 mmHg, temperature = 36.7 °C, saturation = 85% in room air, coarse crackle in lung auscultation, and bilateral pitting edema in his feet. There were no other findings in his physical examination. The patient was in stupor state without eye or verbal contact. He was only responsive to painful stimulus. Pupils were reactive to light and gag reflex was absent. Plantar reflex was normal. The patient had a suprapubic catheter because of benign prostatic hyperplasia for therapeutic purposes since 2 months before admission.

We asked for blood tests and CT scans for the patient. Blood tests showed WBC of 4.1 × 109 (neutrophils 86%, lymphocytes 9.6%), anemia (RBC = 4.34 cells/mcL, hemoglobin level = 8.8 g/dL, hematocrit = 28.8%, hypochromia ++, schistocyte +), thrombocytopenia (platelet = 110,000/mcL), abnormal electrolytes level (sodium = 158 mmol/L, potassium = 3 mmol/L, phosphorus = 5.4 mg/dL, calcium = 7.2 mg/dL), urea of 140 mmol/L, lactate dehydrogenase of 602 U/L, erythrocyte sedimentation rate (ESR) of 20 mm/h, C-reactive protein of 87.41 mg/L, creatinine of 2 mg/dL and serum albumin of 1.6 g/dL and blood sugar of 121 mg/dL.

Brain CT scan was performed, as the patient had LOC, which revealed senile brain atrophy without any abnormal findings.

**Fig. 1:**
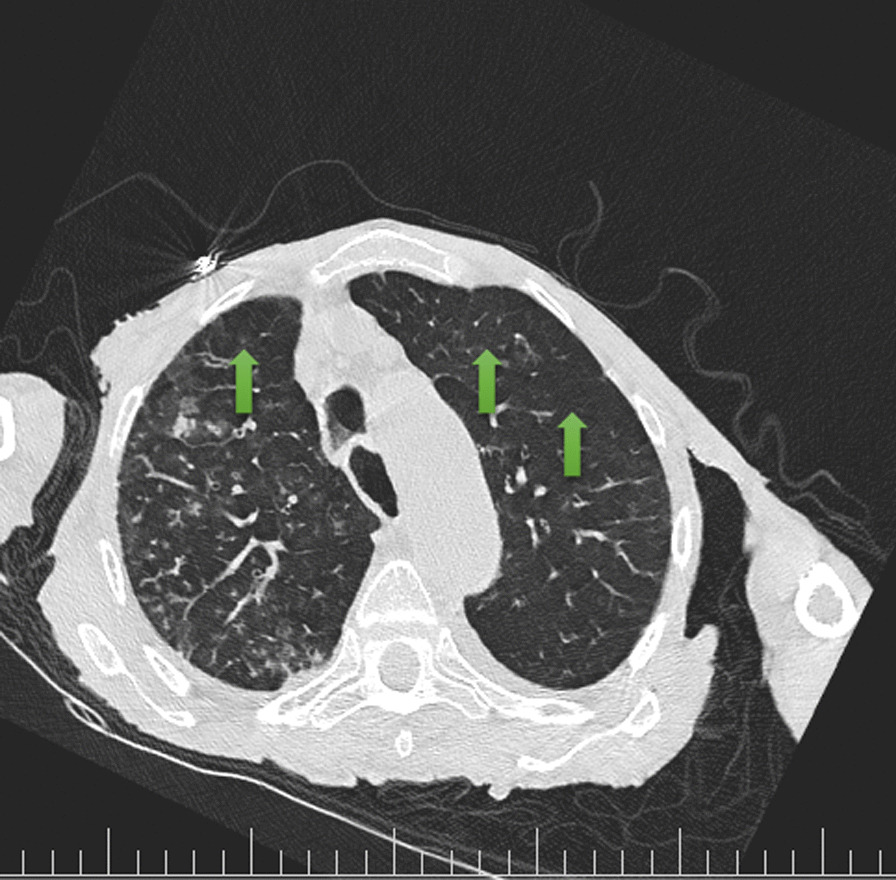
Air trapping in the anterior aspect of both lung fields is identified by green arrows

**Fig. 2:**
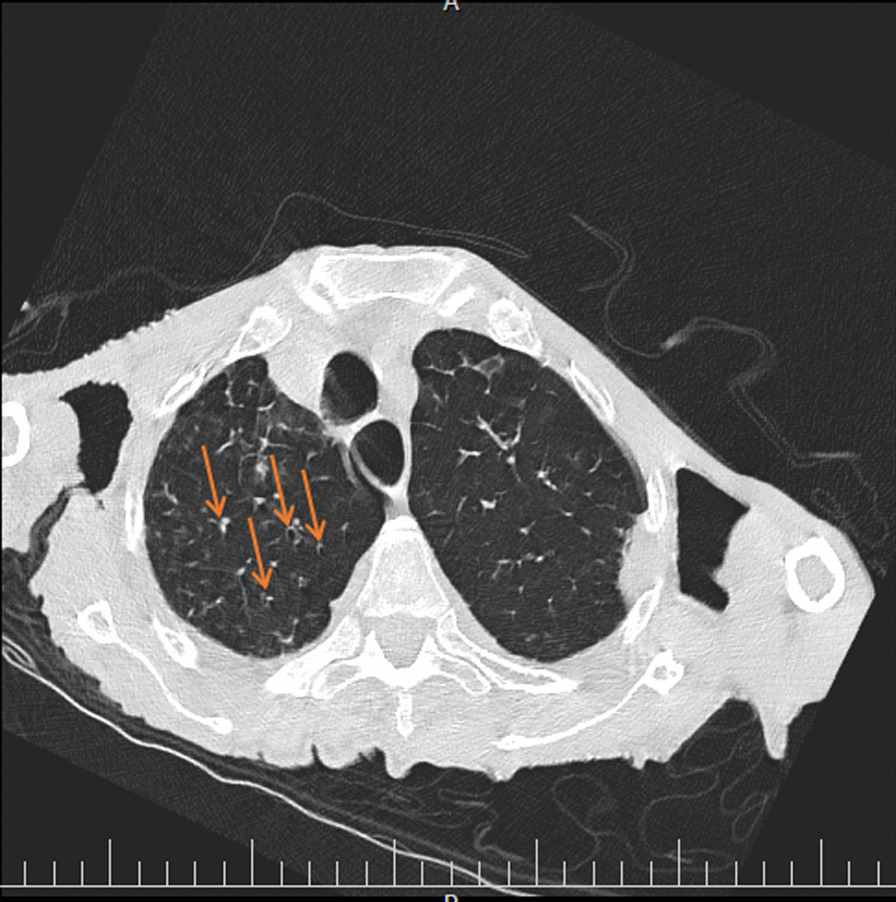
Arrows show right upper lobe involvement and diffuse peribronchial cuffing

**Fig. 3:**
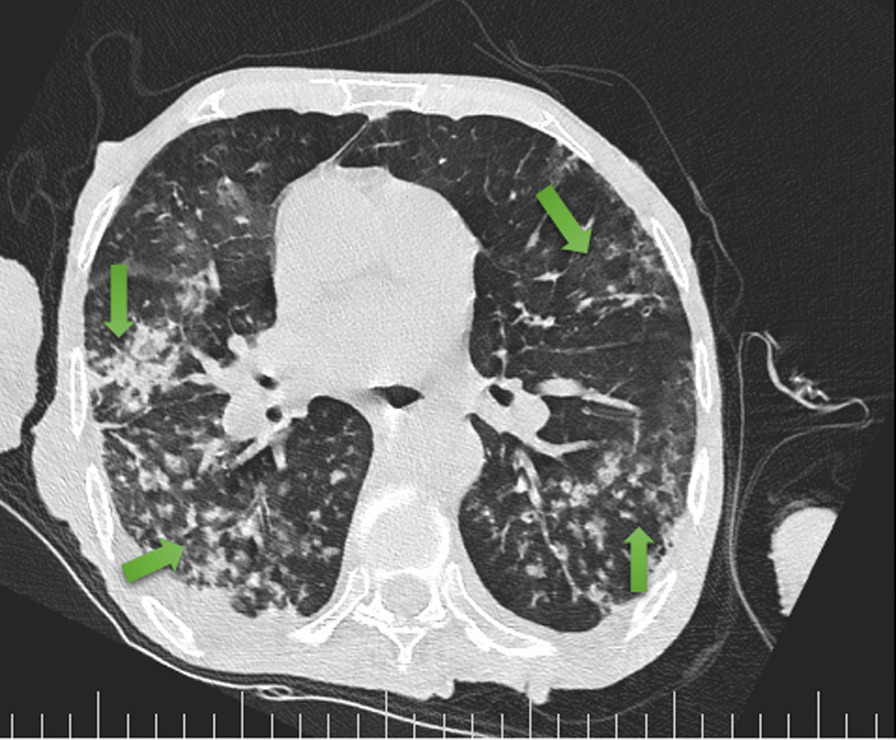
Arrows indicate diffuse patchy ground glass opacity and consolidation

**Fig. 4:**
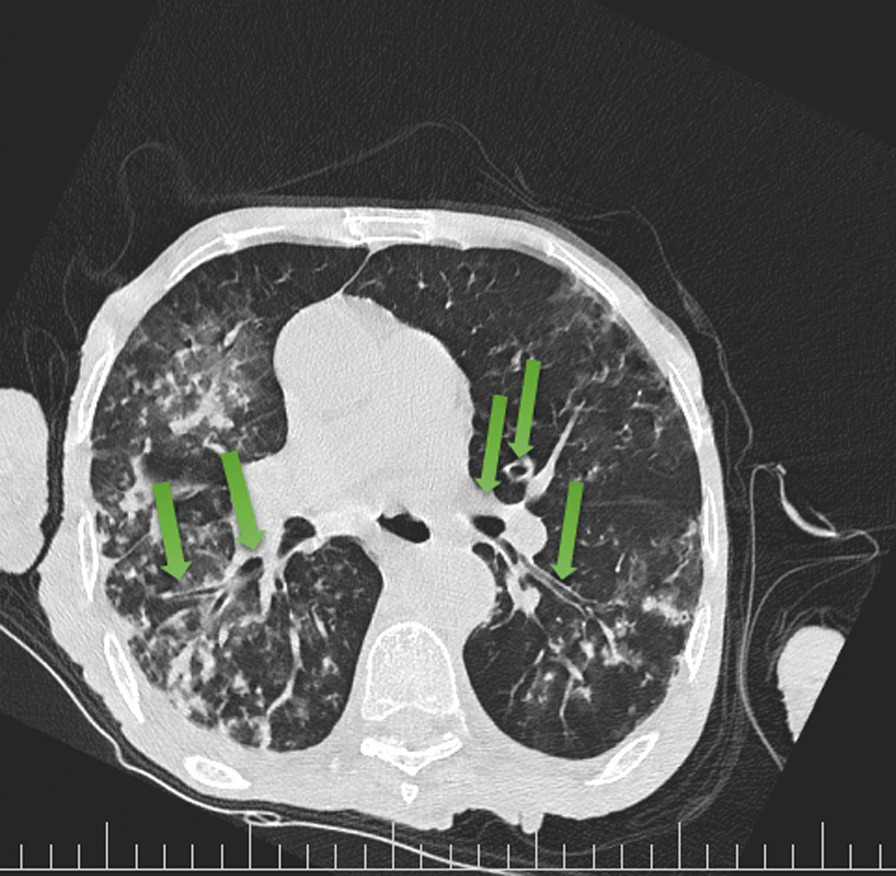
Arrows show diffuse bilateral peribronchial thickening

His chest CT scan showed air trapping in the anterior aspect of both lung fields (Fig. 1), right upper lobe (RUL) involvement and diffuse peribronchial cuffing (Fig. 2), diffuse patchy ground glass opacity (GGO), and consolidation that some of them were subpleural and some of them showed crazy paving appearance because of adding interlobular septal thickening that they could be due to viral infection (Fig. 3). Moreover, diffuse bilateral peribronchial thickening was seen (Fig. 4).

Blood cultures were taken from two different sites which revealed no growth after 48 hours.

During his hospitalization, nasal respiratory viral panel multiplex RT-PCR was taken and revealed co-infection of influenza virus and RSV; therefore sepsis and pneumonia treatment was initiated for the patient including meropenem 1gr infused over 3 hours once a day, vancomycin 1 gr every 72 hours intravenously and oseltamivir 75 mg every 12 hours orally. He also received Albumin twice a day because of decreased level of albumin. Intravenous infusion of 5% dextrose water and half saline was administered according to standard protocols to maintain hydration and balance hypernatremia in the patient.

On the 7th day of his hospitalization the patient had become anuric. Creatinine and blood urea nitrogen (BUN) level increased (creatinine: 4 mg/dL, BUN: 122 mg/dL), venous blood gas test revealed a metabolic acidosis (PH: 7.11, pCO_2_: 18.2, HCO_3_: 8.6) which was resistant to treatment. An urgent surgery consultation was requested for central venous catheter placement for performing dialysis but in the meantime, his blood pressure (BP) dropped and norepinephrine drip was infused. Because of low BP, the patient could not tolerate dialysis. Due to septic shock, he had cardiopulmonary arrest at night, and regardless of intubation and 45 minutes of cardiopulmonary resuscitation, he died.

## Discussion and conclusion

This case emphasizes the importance of accurate diagnosis and prompt intervention when dealing with co-infections involving influenza virus and RSV.

RSV infection was firstly described in children. Therefore epidemiology and burden of the disease among children had been well described [3]. Nowadays in addition to children, prevalence of RSV is increasing in older adults [2, 3].

A systematic review on RSV epidemiology in adults and elderlies discovered that RSV constituted 69.9% and 91.7%, of hospitalization in adults with influenza-like illness and community-acquired pneumonia, respectively; therefor, RSV may be a more significant cause of serious respiratory illness in adults than previously recognized [7].

Influenza virus is single-stranded, enveloped ribonucleic acid (RNA) virus which includes four types A to D. Type A is the most common type that causes seasonal flu and moderate to severe infection [1, 5, 8].

The main transmission routs of both viruses are mucus droplets and contact [9, 10].

Binding of flu and RSV viral genome segments may result in recombinant viruses that are highly pathogenic to populations [11].

Evaluation the prevalence of co-infection between RSV and influenza A virus (IAV) and influenza B virus (IBV) independently in children revealed even when both viruses are circulating in the community, incidence of co-infection of RSV and influenza was significantly less than expected [12]. George *et al.* in a mouse model study of RSV and IAV co-infection have found that among all infection types (including RSV, IAV, RSV + IAV, IAV + RSV), infection with IAV followed by RSV was associated with the highest influenza viral load as well as the most morbidity and mortality rate [1]. Another study in china with human subjects found that mortality was higher in the coinfected (influenza + RSV) patients than RSV-only infected patients although antivirals such as oseltamivir were prescribed. It may suggest that oseltamivir could not decrease mortality risk of RSV and influenza coinfection [13]. These studies show that RSV and influenza co-infection is significant and can be severe; however it is a rare condition.

During COVID-19 pandemic, because of overlapping symptoms of SARS-COV-2 infection with other respiratory infections diagnosis based on clinical findings was difficult for physicians.

Performing multiplex PCR testing through sputum or throat swabs helps to distinguish different pathogens such as influenza A virus, influenza B virus, Adenovirus, RSV, Chlamydia pneumoniae and Mycoplasma pneumoniae from COVID-19 infection [14]. However, this method may be related to multiple factors such as quality of sampling, specimen preservation, and varying concentration of nasopharyngeal at different stages of the disease [15].

Numerous studies have shown that rapid and accurate diagnosis of respiratory infections enhances clinical management by adjusting the indications for isolation, antibiotic, and antiviral treatment due to better analytical performances than rapid diagnostic tests during seasonal IAV, IBV and RSV epidemics [16–18].

Influenza treatment is primarily based on neuraminidase inhibitors (oseltamivir and zanamivir).However, these medications need to be started within 48 hours of symptom onset and are most effective when taken within 24 hours [19]. There are currently no specific guidelines for the treatment of RSV in the adult population [20]. Ribavirin use has been shown to reduce mortality in adults with RSV infection [21].

Stefanska *et al.*, reported that oseltamivir improved conditions of patients with coinfection with influenza and other respiratory viruses (RSV, coronavirus OC43 and parainfluenza) after treatment (22).

In conclusion, simultaneous infection with influenza virus and RSV increase the severity of the disease. In our case, old age, weak immune system and late diagnosis of the disease caused delay in the initiation of appropriate treatment. As a takeaway lesson of this case report, it is necessary to pay attention to viruses which shows similar symptoms during future viral pandemics.

## Data Availability

The data sets used during the current study are available from the corresponding author on reasonable request.

## Abbreviations

RSV: Respiratory syncytial virus
IAV: Influenza A virus
IBV: Influenza B virus
CT: Computed tomography
COVID-19: Coronavirus disease 2019
RNA: Ribonucleic acid
SARS-COV-2: Severe acute respiratory syndrome coronavirus 2
PCR: Polymerase chain reaction
BP: Blood pressure
RUL: Right upper lobe
GGO: Ground glass opacity
